# 成人急性髓系白血病（非急性早幼粒细胞白血病）中国诊疗指南（2023年版）

**DOI:** 10.3760/cma.j.issn.0253-2727.2023.09.001

**Published:** 2023-09

**Authors:** 中华医学会血液学分会白血病淋巴瘤学组

修订要点：

• 初诊急性髓系白血病（AML）增加BCOR、EZH2、SF3B1、SRSF2、STAG2、U2AF1、ZRSR2基因突变的分子学检测。

• AML患者危险度在预后良好组中CEBPA双突变更新为CEBPA bZIP框内突变，预后不良组中增加了BCOR、EZH2、SF3B1、SRSF2、STAG2、U2AF1、ZRSR2基因突变及t（8；16）（p11; p13）/KAT6A::CREBBP和11p15/NUP98基因易位。

• 增加疗效评价标准。

• AML治疗由以年龄为分层主要标准，更新为综合多种因素评估能够耐受化疗为分层主要标准。

• 在可以耐受化疗AML中，增加了化疗联合靶向药物作为初始诱导治疗方案。

• 可检测残留病（MRD）指导分层治疗中给出了MRD分层的监测方法、时间点及阈值。

• 在可以耐受化疗AML巩固治疗中，有FLT3突变的中高危组患者可以在巩固治疗中联合FLT3抑制剂。

• 在可以耐受化疗AML维持治疗中，强调中高危组患者可用去甲基化药物进行维持治疗。

• 不耐受强化疗AML治疗方案增加IDH1和FLT3突变AML可以应用IDH1或FLT3抑制剂的治疗方案。

## 第一部分 初诊患者入院检查及诊断

一、病史采集及重要体征

• 年龄

• 此前有无血液病病史，主要指骨髓增生异常综合征（MDS）、骨髓增殖性肿瘤（MPN）等

• 是否为治疗相关性（包括肿瘤放疗、化疗）

• 有无重要脏器功能不全（主要指心、肝、肾功能等）

• 有无髓外浸润［主要指中枢神经系统白血病（CNSL）、皮肤浸润、髓系肉瘤］

• 有无白血病或者肿瘤家族史，有无遗传代谢性疾病病史

二、实验室检查

• 血常规、血生化、出凝血检查

• 骨髓细胞形态学（包括细胞形态学、细胞化学、组织病理学）

• 免疫分型

• 细胞遗传学（染色体核型），必要时荧光原位杂交（FISH）

• 分子学检测

PML::RARα、RUNX1::RUNX1T1、CBFβ::MYH11、KMT2A重排、BCR::ABL1、C-KIT、FLT3-ITD、NPM1、CEBPA、TP53、RUNX1、ASXL1、BCOR、EZH2、SF3B1、SRSF2、STAG2、U2AF1、ZRSR2、IDH1、IDH2、DNMT3a基因突变，这些检查是AML分型、危险度分层及指导治疗方案的基础[1]–[5]（证据等级1a）。

• 对于有CEBPA、RUNX1、DDX41、GATA2等基因突变的患者，建议进行同一基因的胚系突变检查以除外胚系易感急性髓系白血病（AML）

• 有意愿行异基因造血干细胞移植的患者行HLA配型

三、诊断及分类

AML的诊断标准参照WHO 2016造血和淋巴组织肿瘤分类标准，外周血或骨髓原始细胞≥20％是诊断AML的必要条件。但当患者被证实有克隆性重现性细胞遗传学异常t（8;21）（q22；q22）、inv（16）（p13q22）或t（16；16）（p13；q22）以及t（15;17）（q24;q21）时，即使原始细胞<20％，也应诊断为AML[2]。

2022年WHO更新了造血和淋巴组织肿瘤分类标准（WHO2022）[6]，同时国际上一个由临床、病理及遗传学方面专家组成的小组发布了国际共识分类（International Consensus Classification，ICC）[7]。本指南制定专家组注意到这些国际上新的标准建议，两个最新的国际分类有关AML的诊断标准有部分不同，国内外专家都期待国际上能够统一造血和淋巴组织肿瘤分类标准，我们仍需要进一步评估这些新的标准。因此，在这一版国内AML指南中，我们仍采用WHO 2016造血和淋巴组织肿瘤分类标准。

四、AML的预后和分层因素

1. AML不良预后因素：

• 年龄≥60岁

• 有MDS或MPN病史

• 治疗相关性/继发性AML

• 高白细胞（WBC≥100×10^9^/L）

• 合并CNSL

• 合并髓外浸润（除外肝、脾、淋巴结受累）

2. 细胞遗传学/分子遗传学指标危险度分级：目前国内主要是根据初诊时AML细胞遗传学和分子遗传学的改变进行AML遗传学预后分组，具体分组见表1 [3]–[5],[8]–[9]。

**表1:** 急性髓系白血病（AML）患者的预后危险度

预后等级	细胞遗传学	分子遗传学
预后良好	inv(16)(p13q22)或t(16;16)(p13;q22) /CBFβ::MYH11 t(8;21)(q22;q22)/RUNX1::RUNX1T1	NPM1突变不伴有FLT3-ITD突变，或者伴有低等位基因比FLT3-ITD突变^ad^ CEBPA bZIP框内突变^b^
预后中等	正常核型 t(9;11) (p22;q23)/ MLLT3::KMT2A 其他异常	inv(16)(p13q22)或t(16;16)(p13;q22)伴有C-KIT D816突变^c^ t(8;21)(q22;q22)伴有C-KIT D816突变^c^ NPM1 突变伴有高等位基因比FLT3-ITD突变^d^
预后不良	单体核型 复杂核型(≥3种)，不伴有t(8;21)(q22；q22)、inv(16)(p13q22)或t(16;16)(p13;q22)或t(15;17)（q22;q12） −5 −7 5q− −17或abn(17p) 11q23染色体易位，除外t(9;11) inv(3) (q21q26.2)或t(3;3)(q21q26.2)/GATA2，MECOM(EVI1) t(3q26.2;v)/MECOM(EVI1)-重排 t(6;9) (p23;q34) /DEK::NUP214 t(9;22)(q34.1;q11.2) /BCR::ABL1 11p15/NUP98基因易位 t(8;16)(p11; p13) /KAT6A::CREBBP	TP53突变 RUNX1、ASXL1、BCOR、EZH2、SF3B1、SRSF2、STAG2、U2AF1、ZRSR2突变^e^ 高等位基因比FLT3-ITD突变^de^

注 ^a^ AML患者同时携带不良细胞遗传学异常和NPM1突变为不良预后组。^b^ AML伴CEBPA基因BZIP结构框内突变，包括单基因或双等位基因突变。^c^ RUNX1::RUNX1T1或CBFB::MYH11的AML伴有C-KIT D816突变为预后中等组，其他的突变位点对预后没有影响，归入预后良好组。^d^ 低等位基因比为<0.5，高等位基因比为≥0.5；如没有进行FLT3等位基因比检测，FLT3-ITD阳性应该按照高等位基因比对待。^e^ 这些异常如果发生在预后良好组时，不应作为预后不良的标志。单体核型：两个或两个以上常染色体单体，或一个常染色体单体合并至少一个染色体结构异常，除外不伴结构异常由三倍体形成的复杂核型

## 第二部分 AML（非急性早幼粒细胞白血病）患者的治疗

所有AML患者，在能够参加临床研究的情况下，均建议首选参加临床研究。缺乏临床研究者，可以参照下述建议进行治疗。AML的治疗以化疗、造血干细胞移植及联合新近出现的靶向治疗为主要治疗方法，目前强化疗仍然是可以耐受化疗AML患者的推荐治疗方案。AML患者化疗的耐受性要根据年龄、体力状态、共病等多种因素进行综合评估，且在治疗过程中进行动态评估，调整治疗策略。对于不耐受化疗患者的评估，指南推荐参照Ferrara 2013 标准进行筛选，具体筛选标准见表2。初诊不能耐受强烈治疗的患者经过低强度诱导治疗达完全缓解后，如果可以耐受强化疗，应按照可以耐受强化疗患者的治疗方案选择。

**表2:** 急性髓系白血病不适合进行强化疗标准

1. 年龄超过75岁
2. 伴有充血性心力衰竭或既往有射血分数（EF）≤50％的心肌病史
3. 既往有肺部疾病史，肺一氧化碳弥散量（DLCO）≤65％或第1秒用力呼气容积（FEV1）≤65％，或静止时有呼吸困难或需要吸氧，或任何胸膜肿瘤或未得到控制的肺部肿瘤
4. 正接受透析治疗且年龄大于60岁，或未得到控制的肾脏肿瘤
5. Child B或C级的肝硬化，或伴有转氨酶大幅升高（>正常值的3倍）的肝病且年龄≥60岁，或任何胆管癌，或未控制的肝癌或急性病毒性肝炎
6. 存在抗感染治疗耐药的活动性感染
7. 伴有需要在精神病院、管制机构住院治疗及持续频繁门诊治疗的精神疾病，或存在照顾者不能控制的依赖性认知状态（由专科医师确诊）
8. 与白血病无关的ECOG体能状态评分≥3
9. 医师认为不适合接受常规强化化疗的任何其他合并症

注 满足表中至少一项标准则表明患者不适合进行强化疗

一、可以耐受强化疗AML

（一）诱导治疗

1. 常规的诱导治疗方案：标准剂量阿糖胞苷（Ara-C）100～200 mg·m^−2^·d^−1^×7 d联合去甲氧柔红霉素（IDA）12 mg·m^−2^·d^−1^×3 d或柔红霉素（DNR）60～90 mg·m^−2^·d^−1^×3 d[10]–[12]（证据等级1a）。

2. 含中剂量Ara-C的诱导治疗方案：高三尖杉酯碱（HHT）2 mg·m^−2^·d^−1^×7 d，DNR 40 mg·m^−2^·d^−1^×3 d，Ara-C前4 d为100 mg·m^−2^·d^−1^，第5、6、7天为1 g·m^−2^·12 h^−1^[13]–[14]（证据等级1a）。

3. 其他化疗方案：IA、DA、MA及HA＋蒽环类药物组成的方案，如HAA（HA+阿克拉霉素）、HAD（HA+DNR）等[15]。HHT（或三尖杉酯碱）联合标准剂量Ara-C的方案（HA）。化疗药物推荐使用剂量：标准剂量Ara-C 100～200 mg·m^−2^·d^−1^×7 d。IDA 10～12 mg·m^−2^·d^−1^×3 d、DNR 45～90 mg·m^−2^·d^−1^× 3 d、米托蒽醌（Mitox）6～10 mg·m^−2^·d^−1^×3 d、阿克拉霉素（Acla）20 mg/d×7 d、HHT 2～2.5 mg·m^−2^·d^−1^× 7 d（或4 mg·m^−2^·d^−1^×3 d）。临床工作中可以参照上述方案，具体药物剂量可根据患者情况调整。对于有严重合并症患者，参照老年不耐受强烈化疗患者的治疗方案。

4. 联合靶向药物的治疗方案：研究显示在化疗方案的基础上联合靶向药物可以提高缓解率及MRD转阴比例[16]–[18]，因此，可以酌情考虑在化疗基础上联合靶向药物，中高危组联合维奈克拉（1～2周）；高危组接受标准强化诱导治疗缓解率低于低中危组，亦可采用维奈克拉联合去甲基化药物诱导治疗；FLT3突变患者可以联合FLT3抑制剂；IDH突变患者可以联合IDH抑制剂（证据等级2b）。

（二）诱导治疗后监测

诱导治疗后恢复期（停化疗后第21～28天左右）复查骨髓以评价疗效，根据骨髓情况决定下一步的治疗方案。对于接受标准剂量，特别是低强度诱导治疗的患者，可以在诱导治疗过程骨髓抑制期（停化疗后第7～14天）复查骨髓，根据骨髓原始细胞残留情况，调整治疗方案。

1. 标准剂量Ara-C诱导治疗后监测：

（1）停化疗后第7～14天复查骨髓：

①存在明显的残留白血病细胞（≥10％），可以考虑双诱导治疗，建议方案：

• 标准剂量Ara-C+蒽环或蒽醌类等药物（IDA或DNR、Mitox等）

• 含G-CSF的预激方案（如CAG方案：G-CSF+Ara-C+Acla）

• 等待观察（尤其是处于骨髓增生低下时）

②残留白血病细胞<10％，但无增生低下，可给予双诱导治疗，采用标准剂量Ara-C+IDA或DNR、Mitox等；或等待恢复。

③增生低下，残留白血病细胞<10％时，等待恢复。

（2）停化疗后第21～28天（骨髓恢复）复查骨髓、血象：

①完全缓解，进入缓解后治疗。

②增生低下，残留白血病细胞<10％时，等待恢复。

③骨髓已恢复，未达到完全缓解标准的，按治疗失败对待。

2.含中大剂量Ara-C方案的诱导治疗后监测：停化疗后第21～28天（骨髓恢复）复查骨髓、血象。

（1）完全缓解，进入缓解后治疗。

（2）增生低下，残留白血病细胞<10％时，等待恢复。

（3）骨髓已恢复，未达到完全缓解标准的，按治疗失败对待。

（三）疗效评价标准

完全缓解（CR）：骨髓原始细胞小于5％，外周血原始细胞及髓外病灶消失，中性粒细胞≥1×10^9^/L，PLT≥100×10^9^/L。

完全缓解伴部分血液学恢复（CRh）：骨髓原始细胞小于5％，外周血原始细胞及髓外病灶消失，中性粒细胞≥0.5×10^9^/L，PLT≥50×10^9^/L。

完全缓解伴不完全血液学恢复（CRi）：骨髓原始细胞小于5％，外周血原始细胞及髓外病灶消失，中性粒细胞<1×10^9^/L，或PLT<100×10^9^/L。如果同时使用CRh和CRi，CRi只包括不符合CRh的患者。

骨髓无白血病状态（MLFS）：骨髓原始细胞小于5％，外周血原始细胞及髓外病灶消失，对血象恢复没有要求。

部分缓解：中性粒细胞≥1×10^9^/L，PLT≥100×10^9^/L，骨髓原始细胞百分比下降≥50％，并且原始细胞比例为5％～25％。

（四）完全缓解后治疗的选择

按对化疗的耐受性、遗传学预后危险度及可检测残留病（Measurable residual disease, MRD）相结合分层治疗，MRD既往称微小残留病（Minimal residual disease, MRD）。伴有FLT3突变的中高危组患者可以在巩固治疗中联合FLT3抑制剂。

对化疗的耐受性应进行动态评估，缓解后应再次综合年龄、体力状态、共病等多种因素评估其耐受性。对强化疗耐受好、从化疗中获益大的患者可以积极进行大剂量化疗；对强化疗耐受好、从化疗中获益不大的高危组患者可以积极进行异基因造血干细胞移植；对强化疗耐受差、从化疗中获益不大的患者可以选择新的靶向及免疫治疗策略。

MRD可以采用多参数流式细胞术、定量PCR等方法进行检测。在进行危险度分层时，除按照治疗前遗传学检测结果进行危险度分层外，可根据治疗后MRD检测结果进行动态危险度分层调整。对于MRD持续阳性，或MRD由阴性转为阳性，尤其是巩固治疗完成后MRD阳性的患者，虽然遗传学分层属于预后中低危组，仍然建议进行异基因造血干细胞移植。核心结合因子（CBF）白血病2个疗程化疗后，融合基因下降<3个log，建议行异基因造血干细胞移植，无初诊融合基因表达数据时，以>0.1％为阈值[8],[19]。应用流式细胞术进行MRD检测时，初诊预后低危组2个疗程化疗后，MRD阳性患者，建议行异基因造血干细胞移植；预后中等组1个疗程化疗后，MRD阳性患者，建议行异基因造血干细胞移植。

1. 预后良好组：

（1）多疗程的大剂量Ara-C：大剂量Ara-C（3 g·m^−2^·12 h^−1^，6个剂量），3～4个疗程，单药应用[20]–[21]（证据等级1a）。

（2）其他缓解后治疗方案：

①中大剂量Ara-C（1～2 g·m^−2^·12 h^−1^，6个剂量）为基础的方案：与蒽环/蒽醌类、氟达拉滨等联合应用，2～3个疗程后行标准剂量化疗，总的缓解后化疗周期≥4个疗程[13]–[15],[22]（证据等级1b）。

②2～3个疗程中大剂量Ara-C为基础的方案巩固，继而行自体造血干细胞移植[23]–[26]（证据等级1b）。

③标准剂量化疗（Ara-C联合蒽环/蒽醌类、HHT、鬼臼类等），总的缓解后化疗周期≥6个疗程或标准剂量化疗巩固3～4个疗程后行（或不行）自体造血干细胞移植[26]–[27]（证据等级2b）。

2. 预后中等组：

（1）异基因造血干细胞移植。寻找供者期间行1～2个疗程的中大剂量Ara-C为基础的化疗或标准剂量化疗[28]（证据等级1a）。

（2）多疗程的中大剂量Ara-C。中大剂量Ara-C（1.5～3 g·m^−2^·12 h^−1^，6个剂量），3～4个疗程，单药应用[20]–[21]（证据等级1a）。

（3）2～3个疗程中大剂量Ara-C为基础的巩固治疗后行自体造血干细胞移植[23]–[25],[29]（证据等级1b）。

（4）其他巩固治疗方案：

①中大剂量Ara-C（1～2 g·m^−2^·12 h^−1^，6个剂量）为基础的方案：与蒽环/蒽醌类等药物联合应用，2～3个疗程后行标准剂量化疗，总的缓解后化疗周期≥4个疗程[13]–[15],[22]（证据等级1b）。

②标准剂量化疗（Ara-C联合蒽环/蒽醌类、HHT、鬼臼类等），总的缓解后化疗周期≥6个疗程或标准剂量化疗巩固3～4个疗程后行（或不行）自体造血干细胞移植[27],[29]（证据等级2b）。

3. 预后不良组：

（1）尽早行异基因造血干细胞移植。寻找供者期间行1～2个疗程的中大剂量Ara-C为基础的化疗或标准剂量化疗[28]（证据等级1a）。

（2）无条件移植者予中大剂量Ara-C（1.5～3 g·m^−2^·12 h^−1^，6个剂量），3～4个疗程，单药应用[20]–[21]（证据等级1a）。

（3）其他巩固治疗方案：

①2～3个疗程的中大剂量Ara-C为基础的化疗，或标准剂量化疗巩固，继而行自体造血干细胞移植[23]–[25]（证据等级1b）。

②标准剂量化疗巩固（≥6个疗程）[27]（证据等级1a）。

③维奈克拉联合去甲基化药物（如阿扎胞苷或地西他滨）或者去甲基化药物单药治疗，直至疾病进展[30]–[34]（证据等级2b）。

4. 未进行染色体核型及相关基因等检查、无法进行危险度分层者：参考预后中等细胞遗传学或分子异常组患者治疗。若诊断时WBC≥100×10^9^/L，则按预后不良组治疗（证据等级5）。

（五）维持治疗

1. 经过诱导和巩固治疗后，中高危组患者可用去甲基化药物（如阿扎胞苷或地西他滨）进行维持治疗，直至疾病进展[35]–[36]（证据等级1b）。

2. 异基因造血干细胞移植后，视复发风险及造血重建状态，FLT3-ITD阳性患者可以选择FLT3抑制剂维持，其他患者可以选择去甲基化药物维持治疗[37]–[38]（证据等级1b）。

二、不耐受强化疗AML

（一）诱导治疗

1. 低强度治疗方案：

（1）维奈克拉（100 mg第1天，200 mg第2天，400 mg第3～28天）联合阿扎胞苷（75 mg·m^−2^·d^−1^，7 d）或地西他滨（20 mg·m^−2^·d^−1^，5 d），每28 d 1个周期[33]–[34]（证据等级1a）。

（2）阿扎胞苷（75 mg·m^−2^·d^−1^，7 d）或地西他滨（20 mg·m^−2^·d^−1^，5 d）[30]–[32]。

（3）阿扎胞苷或地西他滨联合小剂量化疗；小剂量化疗±G-CSF（如小剂量Ara-C为基础的方案CAG、CHG、CMG等，C-阿糖胞苷、A-阿克拉霉素、H-高三尖杉酯碱、M-米托蒽醌）[39]–[40]（证据等级2b）。

2. IDH1突变AML：除前述治疗方案，可以选择艾伏尼布（500 mg，第1～28天）联合阿扎胞苷（75 mg·m^−2^·d^−1^，7 d），每28 d 1个周期（证据等级1a）或艾伏尼布单药治疗[41]（证据等级2b）。

3. FLT3突变AML：除前述治疗方案，可以选择吉瑞替尼（120 mg，第1～28天）联合维奈克拉（100 mg第1天，200 mg第2天，400 mg第3～28天），每28 d 1个周期，或吉瑞替尼（120 mg，第1～28天）联合阿扎胞苷（75 mg·m^−2^·d^−1^，7 d），每28 d 1个周期[42]–[43]（证据等级2b）。

4. 支持治疗。

（二）完全缓解后的治疗选择

1. 继续前期的低强度治疗方案。

2. 对于预后良好患者，达到完全缓解后，能够耐受标准剂量化疗，可以按照可耐受强化疗AML部分提供的方案进行治疗，包括减剂量/减毒性预处理方案的造血干细胞移植。

三、AML患者中枢神经系统白血病（CNSL）的预防和治疗

AML患者CNSL的发生率远低于急性淋巴细胞白血病（ALL），一般不到3％。参考NCCN的意见，在诊断时对无症状的患者不建议常规行腰椎穿刺（腰穿）检查。有头痛、精神症状、感觉异常的患者应先行影像学检查（CT/MRI），排除神经系统出血或占位。这些症状也可能由于白细胞淤滞引起，可通过白细胞分离去除等降低白细胞计数的措施解决。若体征持续存在、无颅内出血的证据，可在纠正出凝血紊乱和血小板支持下行腰穿。脑脊液中发现白血病细胞者，应在全身化疗的同时鞘内注射（鞘注）Ara-C（40～50 mg/次）和（或）甲氨蝶呤（MTX，5～15 mg/次）+地塞米松（5～10 mg/次）。若症状持续存在，脑脊液无异常，应复查放射学检查及脑脊液。

（一）诊断时有神经系统症状

首先应进行CT/MRI检查，除外颅内出血或占位。

1. 没有发现颅内/脊髓肿块者，进行腰穿及脑脊液检查。

（1）脑脊液正常者：观察。如果症状持续存在，可以再次腰穿检查。

（2）脑脊液发现白血病细胞者：鞘注化疗药物（2次/周）直至脑脊液正常，以后每周1次×4～6周。

2. 发现颅内/脊髓肿块或颅压增高者：建议先行放射治疗；然后鞘注药物（2次/周）直至脑脊液正常，以后每周1次×4～6周。

（二）无神经系统症状，CR_1_后腰穿筛查脑脊液发现白血病细胞者

2次/周鞘注化疗药物直至脑脊液正常，以后每周1次×4～6周。若患者接受大剂量Ara-C治疗，应于治疗完成后复查脑脊液（证实脑脊液正常）；也可以配合腰穿、鞘注，至脑脊液恢复正常。

（三）无神经系统症状，CR_1_后腰穿筛查脑脊液正常者

已达完全缓解的患者，建议行腰穿及预防性鞘注，进行CNSL的筛查。无CNSL患者建议进行4次鞘注治疗。特别是对治疗前WBC≥40×10^9^/L或单核细胞白血病（M_4_和M_5_）、FLT3-ITD、t（8;21）/ RUNX1::RUNX1T1、inv（16）及治疗过程中有颅内出血患者。

四、特别说明

在AML的整个治疗过程中应特别注意化疗药物的心脏毒性问题，注意监测心功能（包括心电图、心肌酶、超声心动图等）。DNR的最大累积剂量550 mg/m^2^。对于具有活动性或隐匿性心血管疾病、目前或既往接受过纵隔/心脏周围区域放疗、既往使用其他蒽环类或蒽二酮类药物治疗、同时使用其他抑制心肌收缩功能药物或具有心脏毒性药物（如曲妥珠单抗）的患者，累积剂量一般不超过400 mg/m^2^。IDA的最大累积剂量290 mg/m^2^，Mitox的最大累积剂量160 mg/m^2^。计算累积剂量时还应考虑整个治疗周期的持续时间、同类药物（如不同的蒽环类药物）的使用情况。
